# In Nature's School

**Published:** 1889-08-10

**Authors:** 


### The Doctor's Pulpit.

### IN NATURE'S SCHOOL.

### Thistledown.

Driving from Canterbury to Folkestone last summer, after a day spent in the venerable cathedral and at other places of interest in the city, a party of modern pilgrims had their attention called to enormous quantities of what appeared to be fine woolly or silky fluff lying by the roadside. One of their number, who possessed a little botanical knowledge, pronounced the "fluff" to be thistledown, which had been blown into the road from the neighbouring fields, and was there waiting until another wind should distribute as seed what the one wind had, with so little evident purpose, industriously gathered together. Every child can find out "what o'clock it is" by puffing away the fluff from a seeding dandelion. It is not every child nor every grown-up person who knows that Nature has provided the seed of the dandelion, thistle, and other kindred plants with the "fluff" for no other purpose but that it may be caught by the wind or the child's breath and distributed to all kinds of places where soil or root-hold may be found. That is one of the commonest methods of seed-sowing known to Nature, and one of the most effectual. The universal and often unwelcome presence of the thistle and dandelion families, and their congeners, is proof positive of its unfailing success.

The seed of the thistle is exceedingly small, and with its attached "fluff" is one of the lightest and, to all appearances, most inconsequent organisms which can well be imagined. It is not only a "trifle light as air," but even lighter than air, and entirely at the mercy of the most frivolous and purposeless wind-puffs. So long as it can be kept away from the soil, so long is it powerless to develop any one of the remarkable potentialities that lie stored up in its diminutive form. But let it once find a lodgment in the soil, and it will soon sink a root many inches deep, and send up a prickly stem three or four feet high, with spreading branches and succulent leaves. Not only so, but one stem will produce dozens of composite flowers, and each flower scores of distinct florets, until at seeding time not only shall the inconsequent piece of fluff have become a plant almost large enough to be called a shrub, but it shall be the parent of a thousand other little pieces of fluff, each as full of potential life and reproductive energy as itself. The life-history of the common thistle is a wonderful and significant thing.

There is a point in this little scientific drama which every keen-minded man will at once appreciate—namely, this: that until the thistledown finds roothold it is one of the most inconsequent of natural things. But once it has got itself firmly established in the soil it becomes a plant that must be reckoned with, both in appearance, in prickles, and in reproductive energy. Is there a man or a woman of experience anywhere who does not know one or two or a dozen of his fellow creatures, who for want of native energy to take root are yet in the stage of thistledown, and who seem likely to remain in that utterly inconsequent condition to the end of their days? There are thousands of men and women who have excellent undeveloped powers, who yet never do anything at all worth the doing. No doubt they will say that they are like the thistledown lying by the roadside, still waiting for the fortunate wind which is to waft them to a suitable rooting place.

It may be at once freely admitted that each individual human being has a right to a "thistledown" stage; as much right, in fact, as the thistledown itself. Childhood and early youth are periods when careless freedom is not only enjoyable, but proper. No wise parent likes to see too grave a face in boy or girl. We cannot put "old heads on young shoulders," nor should we wish. But if even thistledown be possessed of proper spirit it will much prefer seeking a roothold and developing into a thistle to being made use of by tramps in the stuffing of their dirty bedding, which is one of the base uses to which "unattached" thistledown may be put. Similarly, human beings who desire to know by experience what health, wealth, and happiness mean, must, at the proper age, find some means of "rooting" themselves in wholesome and permanent occupation. Human creatures have many advantages over the fluffy down of the thistle or the dandelion. The latter must wait until some casual wind shall carry it and drop it into a suitable and favouring soil. But men and women, if they have sufficient force of character, can look about them and seek "rooting" places for themselves.

Unhappily, there are many who, with every advantage of birth, of money, and of education, do not seem to have sufficient energy of character to find any kind of soil, however poor, in which they may root themselves. If a lucky wind happens to catch them up and drop them into a favourable situation they may do fairly well; but the making of successful efforts on their own behalf seems to be out of the question. It cannot be said that these creatures deserve much consideration. On the contrary, they are apt to exhaust the patience even of their friends very considerably. Instead of recognising the responsibility of acting for themselves, and seeking out such a sphere of duty as may be compatible with their powers, they will fly about from one friend to another, asking advice of all, taking the advice of none, but constantly trying to put the blame of their ill success upon any shoulders but their own. Of course, if such men and women really were irresponsible and brainless thistledown, no doubt Nature would have provided them with sufficient "fluff" to enable the wind to lay hold of them and carry them "somewhere." But being merely shallow-brained and feeble-willed human creatures, they have to lie still on the road-side in helpless poverty and inconsequence if they will not brace up the loins of their mind and do something to help themselves.

Health, for such poor specimens of humanity as these, is impossible. They have not energy enough to seek out and settle down to suitable occupations, but only enough to flutter about like inconsequent moths, and burn themselves at all sorts of convenient and inconvenient candles, blaming other people meanwhile for the strange and astonishing misfortunes which befall them. Nature never intended them to act the part of thistledown, but of rational, purposing, and doing creatures. The man who, having reached mature age, has not resolution enough to choose and follow a definite path for himself need not expect from fortune anything but rebuffs and contempt.

As for health, the inconsequent man never knows when he is well and when he is ill. Having no single thing to do in life except to consider his own changes of feeling and condition, he soon attaches an exaggerated importance to every trifle that affects his own body or mind; he loses all sense of proportion, and becomes of no more real account in the world than the fluffy seed of the thistle which the child blows away on his walk home from school. Nature has a perpetual quarrel with him because he has frustrated her purpose by abdicating his will and becoming less than a man. Until he reassumes the kingdom of his manhood, neither health nor happiness are possible. Of all worthy gifts which life has to offer, health and happiness are the chief. But the greatest gifts demand the highest qualities in him who could secure them. He who cannot resolve upon an effort which shall be persistently steady and resolutely strong may as well give up the battle in despair.

